# Central Nervous System Infection with Borna Disease Virus Causes Kynurenine Pathway Dysregulation and Neurotoxic Quinolinic Acid Production

**DOI:** 10.1128/JVI.00673-17

**Published:** 2017-06-26

**Authors:** Simone Formisano, Mady Hornig, Kavitha Yaddanapudi, Mansi Vasishtha, Loren H. Parsons, Thomas Briese, W. Ian Lipkin, Brent L. Williams

**Affiliations:** a Center for Infection and Immunity, Mailman School of Public Health, Columbia University, New York, New York, USA; b Department of Epidemiology, Mailman School of Public Health, Columbia University, New York, New York, USA; c Committee on the Neurobiology of Addictive Disorders, The Scripps Research Institute, La Jolla, California, USA; d Department of Pathology and Cell Biology, College of Physicians and Surgeons, Columbia University, New York, New York, USA; e Department of Neurology, College of Physicians and Surgeons, Columbia University, New York, New York, USA; Instituto de Biotecnologia/UNAM

**Keywords:** Borna disease virus, central nervous system, kynurenine, neonatal, neurodegeneration, quinolinic acid, tryptophan

## Abstract

Central nervous system infection of neonatal and adult rats with Borna disease virus (BDV) results in neuronal destruction and behavioral abnormalities with differential immune-mediated involvement. Neuroactive metabolites generated from the kynurenine pathway of tryptophan degradation have been implicated in several human neurodegenerative disorders. Here, we report that brain expression of key enzymes in the kynurenine pathway are significantly, but differentially, altered in neonatal and adult rats with BDV infection. Gene expression analysis of rat brains following neonatal infection showed increased expression of kynurenine amino transferase II (KATII) and kynurenine-3-monooxygenase (KMO) enzymes. Additionally, indoleamine 2,3-dioxygenase (IDO) expression was only modestly increased in a brain region- and time-dependent manner in neonatally infected rats; however, its expression was highly increased in adult infected rats. The most dramatic impact on gene expression was seen for KMO, whose activity promotes the production of neurotoxic quinolinic acid. KMO expression was persistently elevated in brain regions of both newborn and adult BDV-infected rats, with increases reaching up to 86-fold. KMO protein levels were increased in neonatally infected rats and colocalized with neurons, the primary target cells of BDV infection. Furthermore, quinolinic acid was elevated in neonatally infected rat brains. We further demonstrate increased expression of KATII and KMO, but not IDO, *in vitro* in BDV-infected C6 astroglioma cells. Our results suggest that BDV directly impacts the kynurenine pathway, an effect that may be exacerbated by inflammatory responses in immunocompetent hosts. Thus, experimental models of BDV infection may provide new tools for discriminating virus-mediated from immune-mediated impacts on the kynurenine pathway and their relative contribution to neurodegeneration.

**IMPORTANCE** BDV causes persistent, noncytopathic infection *in vitro* yet still elicits widespread neurodegeneration of infected neurons in both immunoincompetent and immunocompetent hosts. Here, we show that BDV infection induces expression of key enzymes of the kynurenine pathway in brains of newborn and adult infected rats and cultured astroglioma cells, shunting tryptophan degradation toward the production of neurotoxic quinolinic acid. Thus, our findings newly implicate this metabolic pathway in BDV-induced neurodegeneration. Given the importance of the kynurenine pathway in a wide range of human infections and neurodegenerative and neuropsychiatric disorders, animal models of BDV infection may serve as important tools for contrasting direct viral and indirect antiviral immune-mediated impacts on kynurenine pathway dysregulation and the ensuing neurodevelopmental and neuropathological consequences.

## INTRODUCTION

Borna disease virus (BDV) is a nonsegmented, negative-sense, single-stranded RNA virus belonging to the Bornaviridae family in the order Mononegavirales (1, 2). In both experimental and natural infection of warm-blooded animals, clinical manifestations of Borna disease range from hyperactivity, anxiety, aggression, and movement and posture disorders to abnormal social behaviors and cognitive impairments. However, symptoms vary depending on factors such as animal species and strain, genetic background, immunocompetence, age at infection, virus strain, and route and dose of infection. Infection in the rat is the most commonly studied experimental model of BDV pathogenesis because behavioral symptoms closely resemble those seen in natural infection of horses and ungulates (3–5). Infection of adult Lewis rats results in a biphasic neurological disease. The acute phase occurs approximately 2 to 4 weeks postinfection (p.i.), during which time a severe meningoencephalitis develops, characterized by infiltrating CD4^+^ T cells, CD8^+^ T cells, plasma cells, massive neuronal destruction, and hyperactive-aggressive behavior (6, 7). In the chronic phase, beginning 6 to 8 weeks p.i., immune cells recede from the central nervous system (CNS) over a period of weeks. Despite resolution of inflammation, virus persists in the CNS, and rats exhibit stereotyped motor behaviors, dystonias, and dyskinesias thought to be associated with alterations in the dopamine system (8, 9). However, the masking effects of the cellular antiviral immune response in the adult rat infection model hinder the identification of BDV-specific effects on neuronal pathways. While CNS inflammation is an important element underlying the development and progression of some neurodegenerative disorders, frank encephalitis is an uncommon finding. Thus, animal models without prominent inflammatory effects may unveil important biological mechanisms leading to neurotoxicity, providing greater utility and generalizability.

In contrast with adult infection, neonatal Borna disease (NBD) in the rat results in lifelong viral persistence, characterized by an almost complete lack of inflammation in the CNS (10, 11). NBD provides a unique model for studying virus-induced neurodevelopmental damage and subsequent neurobehavioral disturbances (12). NBD causes hyperactivity, stunted growth, learning deficiencies, altered taste preference, and loss of hippocampal dentate gyrus granule cells, cerebellar Purkinje cells, and cortical neurons (11, 13–15). While NBD is associated with abnormal development of brain monoaminergic systems in the frontal cortex, cerebellum, and hippocampus (16), the cellular and molecular mechanisms by which these disturbances evolve in the absence of CNS inflammation are unclear. Although BDV is noncytolytic *in vitro*, neuronal loss in the hippocampus, neocortex, and cerebellum are hallmark features of NBD (17–19). While it is suggested that immune cell antiviral responses mediate brain cell destruction in adult BDV-infected rats, few studies have addressed specific pathways regulating neurodegeneration in NBD. Our previous studies have implicated a role for activation of the endoplasmic reticulum stress response, poly(ADP-ribose) polymerase 1 (PARP-1) and caspase-3 activation, and neuronal zinc accumulation as distinct mechanisms associated with neurodegeneration in NBD (17, 20, 21). Despite different immunological responses, the potential for overlap in BDV-induced alterations in neuronal pathways between the adult and NBD rat models has not been assessed.

**FIG 1:**
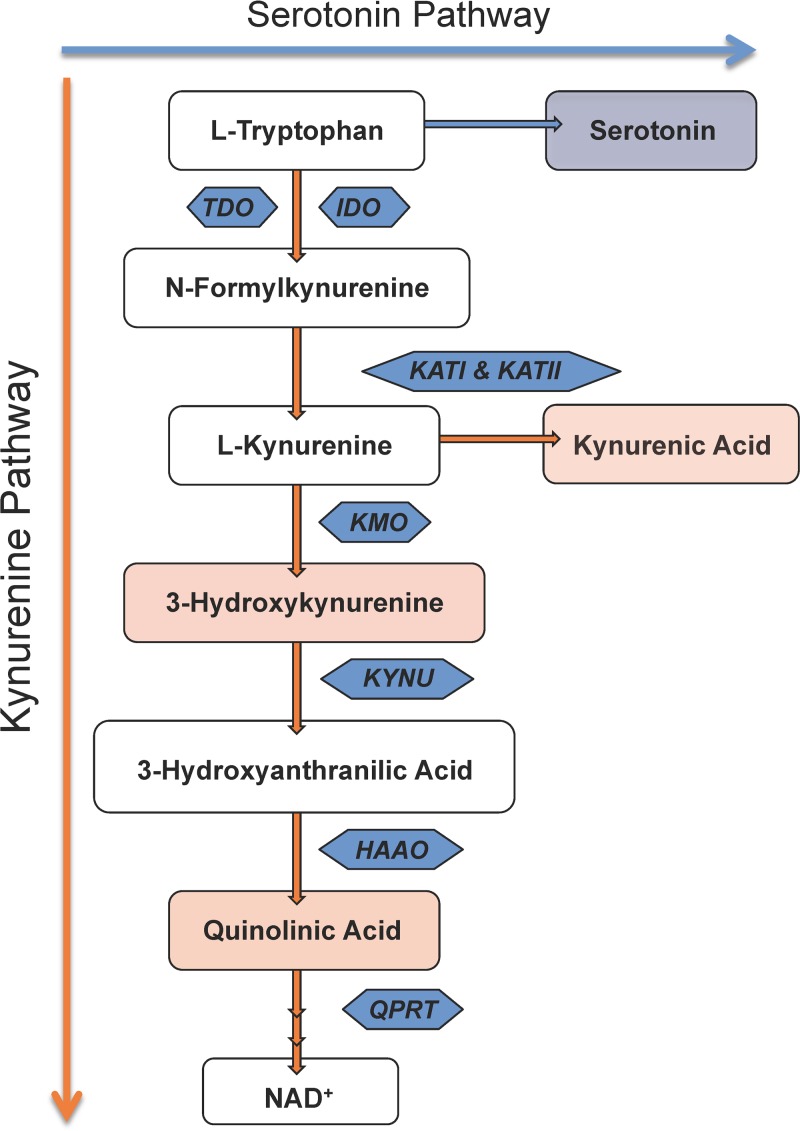
The kynurenine pathway of tryptophan degradation. Several neuroactive metabolites are derived from tryptophan, including the neurotransmitter serotonin and kynurenine pathway metabolites. Along the kynurenine pathway, tryptophan is degraded into three major neuroactive metabolites: 3-hydroxykynurenine, quinolinic acid, and kynurenic acid (pink). Enzymes mediating the degradation of tryptophan along the kynurenine pathway at each intermediary metabolic step are shown in blue. TDO, tryptophan-2,3-dioxygenase; IDO, indoleamine-2,3-dioxygenase; KATI, kynurenine aminotransferase I; KATII, kynurenine aminotransferase II; KMO, kynurenine-3-monooxygenase; KYNU, kynureninase; HAAO, 3-hydroxyantrhanilic acid oxidase; QPRT, quinolinate phosphoribosyl transferase.

Some infections may be associated with increased risk of neuropsychiatric outcomes (22–25). Furthermore, several infectious agents have also been shown to modulate the kynurenine pathway of tryptophan degradation, a pathway that leads to the production of the neuroactive metabolites kynurenic acid (KYNA), 3-hydroxykynurenine (3-HK), and quinolinic acid (QUIN) (Fig. 1). KYNA is an endogenous glutamate and α7 nicotinic acetylcholine receptor antagonist (26–28); 3-HK is a hydroxyl radical generator (29, 30); and QUIN is a potent neural toxin, acting as an *N*-methyl-d-aspartate (NMDA) receptor agonist and free radical generator (31–33). *In vivo* animal models of measles, herpesvirus, Toxoplasma, and influenza virus infection, as well as studies of HIV-1-infected patients, have shown that psychiatric complications and cognitive and behavioral impairments are associated with aberrant tryptophan metabolism and abnormal production of kynurenine pathway metabolites (34–41). Dysregulation of the kynurenine pathway has also been observed in a wide range of human neurodegenerative and neuropsychiatric disorders, including Alzheimer's disease, Huntington's disease, multiple sclerosis, epilepsy, and schizophrenia (39, 42–44).

In this study, we investigated the effect of BDV infection on the kynurenine pathway of tryptophan degradation. We assessed the effects of BDV infection on the expression of key enzymes of the kynurenine pathway in two rat models characterized by differential immunological responses: the NBD model, wherein a persistent, tolerant infection develops, and the immunocompetent adult infection model, wherein BDV induces an immune-mediated meningoencephalitis. Finally, we investigated whether disturbances in this pathway precipitated increased CNS production of the neurotoxin QUIN in NBD that may contribute to regional loss of neurons in this model.

## RESULTS

### Quantitation of BDV RNA and astrogliosis in NBD and adult BDV-infected rat brains.

**FIG 2:**
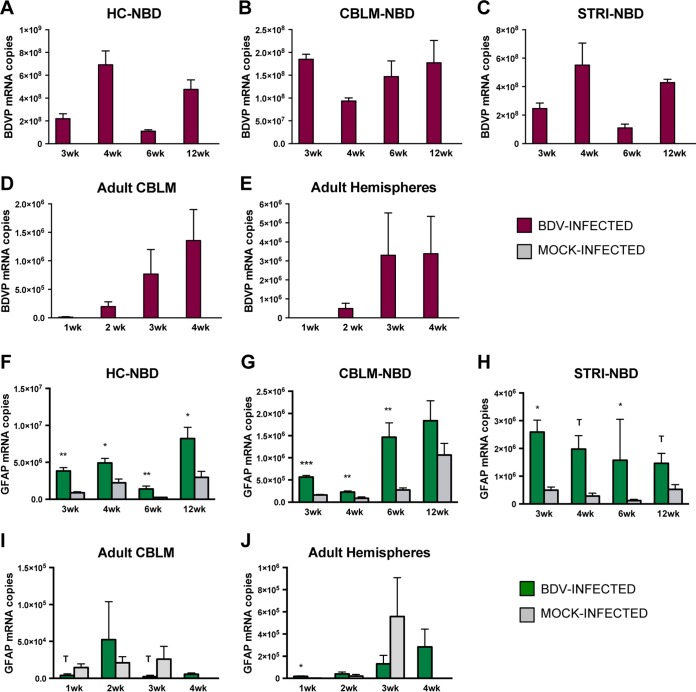
BDV P gene and GFAP mRNA quantitation in NBD and adult BDV-infected rat brains by real-time PCR. (A to C) BDV P RNA levels at 3, 4, 6, and 12 weeks in NBD and neonatal mock-infected rat HC, CBLM, and STRI. (D and E) BDV P RNA levels at 1, 2, 3, and 4 weeks in adult BDV-infected and adult mock-infected rat CBLM and hemispheres. Note that, as expected, no BDV P RNA transcripts were detected in mock-infected rats. (F to H) GFAP expression at 3, 4, 6, and 12 weeks in NBD and neonatal mock-infected rat HC, CBLM, and STRI. (I and J) GFAP expression at 1, 2, 3, and 4 weeks in adult BDV-infected and adult mock-infected rat CBLM and hemispheres. Note that at 4 weeks in the adult model only BDV-infected rats were assessed. *, *P* < 0.05; **, *P* < 0.01; ***, *P* < 0.001; T, *P* < 0.10 (Student's *t* test). wk, weeks.

Previous studies have reported regional preference and time-dependent replication of BDV in rat brains (12, 45–47). In order to assess the load and distribution of BDV in brains of NBD and adult BDV-infected rats, we quantitated RNA transcripts of BDV phosphoprotein (P) in hippocampus (HC), cerebellum (CBLM), and striatum (STRI) of NBD rats at the ages of 3, 4, 6 and 12 weeks, as well as in the CBLM and hemispheres of adult BDV-infected rats at 1, 2, 3, and 4 weeks p.i. In NBD, BDV P was detected at high copy numbers (≥10^8^ copies) in all three brain regions and at each of the four time points (Fig. 2A to C). Consistent with previous findings suggesting preferential replication within the limbic system (48), BDV P RNA copy numbers were higher in the HC and STRI than in the CBLM, especially at 4 weeks. BDV P transcript levels followed a bimodal pattern in the HC and STRI that was not observed in the CBLM. In the HC (Fig. 2A), BDV P levels increased from 3 to 4 weeks (3.2-fold increase relative to week 3), and then levels dropped again by 6 weeks (6.3-fold decrease) and increased again by 12 weeks (4.3-fold increase relative to the week 6 level). Similarly, in the STRI (Fig. 2C), BDV P transcripts increased from 3 to 4 weeks (2.2-fold increase relative to the week 3 level), dropped from 4 to 6 weeks (5-fold decrease relative to the week 4 level), and increased again from 6 to 12 weeks (3.9-fold increase relative to the week 6 level). Fluctuating BDV load could be reflective of the timing of regional neuronal death in the NBD model. In the NBD HC, apoptotic loss of dentate gyrus granule cells, which are a target of BDV infection, begins at around 3 weeks, peaks at around 4 weeks (the time point at which we see the highest levels of viral transcripts), and results in the complete destruction of the granule cell layer by 6 weeks (12). Thus, the progressive loss of these BDV target cells between 4 and 6 weeks could account for our observed fluctuations in BDV loads, which appear to rebound by 12 weeks. Less is known about the nature and timing of neural damage in the striatum, but it is nonetheless intriguing that the timing of BDV P transcript fluctuations is similar to that observed in the HC. In the CBLM of NBD rats (Fig. 2B), there was a moderate decrease in BDV P transcripts at 4 weeks (<2-fold decrease relative to levels at 3, 6, and 12 weeks), correlating with the beginning of Purkinje cell depletion in CBLM in this model (12).

In contrast to the NBD model, in adult BDV-infected rats, BDV P transcript copy numbers were typically lower by 2 to 3 orders of magnitude at the same time points p.i. (3 and 4 weeks p.i., 10^5^ to 10^6^ copies in adult rats versus 10^8^ copies in NBD rats) (Fig. 2D and E). The overall lower viral load detected in the adult BDV-infected rats likely derive from their competent immune status and directed responses against BDV. It has also been suggested that BDV preferentially targets brain regions undergoing substantial postnatal development, which could be an additional factor contributing to these differences (49). The moderate decrease in BDV load that was observed in the CBLM of NBD rats between 3 and 4 weeks (Fig. 2B) was not observed in the adult BDV-infected rat CBLM (Fig. 2D), where viral transcripts increased progressively from 1 to 4 weeks. BDV P was not detected in any of the mock-infected animals by real-time PCR.

Astrocytes are also targets of BDV infection and become activated in response to BDV. It has further been suggested that astrocytes play an important role in microglial activation (50). In order to quantitate the degree of reactive astrogliosis in NBD and adult BDV-infected rats, we have quantitated glial fibrillary acidic protein (GFAP) mRNA by real-time PCR. Consistent with previous reports in NBD rats demonstrating evident reactive astrogliosis by 3 weeks (12), GFAP transcripts were increased in the HC (Fig. 2F), CBLM (Fig. 2G), and STRI (Fig. 2H) of NBD rats compared to levels in neonatal mock-infected rats at 3 weeks (4.33-fold increase, *P* = 0.003; 3.5-fold increase, *P* = 0.003; 5.21-fold increase, *P* = 0.011; respectively). In fact, GFAP transcripts were elevated in NBD rats in each of the three brain regions and at each of the four time points compared to levels in mock-infected rats (fold increases ranged from 2.2 to 12.6), except in the CBLM at 12 weeks.

The evidence for widespread astrogliosis observed in NBD rats was not apparent in adult BDV-infected rats in either the CBLM (Fig. 2I) or hemispheres (Fig. 2J). In the CBLM of adult BDV-infected rats, there was a trend toward decreased GFAP transcripts at 1 week (3.77-fold decrease; *P* = 0.084, Student's *t* test) and 3 weeks (11.33-fold decrease; *P* = 0.083, Student's *t* test) compared to levels in mock-infected rats, and GFAP expression remained low at 4 weeks in BDV-infected rats (Fig. 2I). In the adult BDV-infected rat hemispheres, there was only a modest and transient increase in GFAP transcripts at 1 week (*P* = 0.042, Student's *t* test), with no significant differences found at later time points (Fig. 2J).

### Quantitation of IDO transcripts in NBD and adult BDV-infected rat brains.

**FIG 3:**
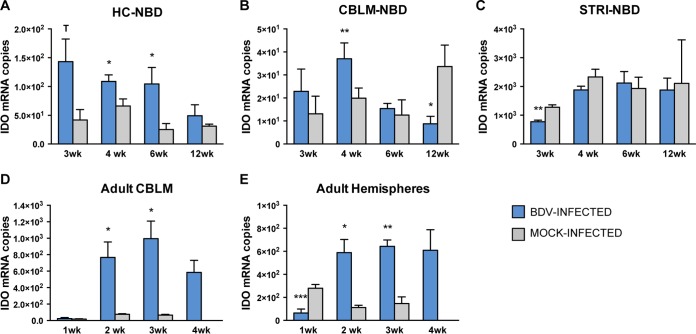
IDO mRNA expression in NBD and adult BDV-infected rat brains by real-time PCR. (A to C) IDO expression at 3, 4, 6, and 12 weeks in NBD and neonatal mock-infected rat HC, CBLM, and STRI. (D and E) IDO expression at 1, 2, 3, and 4 weeks in adult BDV-infected and adult mock-infected rat CBLM and hemispheres. Note that at 4 weeks in the adult model only BDV-infected rats were assessed. *, *P* < 0.05; **, *P* < 0.01; ***, *P* < 0.001; T, *P* < 0.10 (Student's *t* test).

As indoleamine 2,3-dioxygenase (IDO) is the rate-limiting enzyme of the kynurenine pathway, an increase in its expression can shunt tryptophan degradation along the kynurenine pathway (51). Therefore, we quantitated IDO transcripts in the HC, CBLM, and STRI of NBD rats at the ages of 3, 4, 6, and 12 weeks. IDO transcript levels were modestly increased in NBD HC at 4 weeks (1.65-fold increase; *P* = 0.033, Student's *t* test) and 6 weeks (4.17-fold increase; *P* = 0.032, Student's *t* test) compared to levels in neonatal mock-infected HC (Fig. 3A). IDO transcript levels were also increased at 4 weeks (1.86-fold increase; *P* = 0.003, Student's *t* test) but decreased at 12 weeks (3.85-fold decrease, *P* = 0.036, Student's *t* test) in NBD CBLM compared to levels in neonatal mock-infected CBLM (Fig. 3B). Decreased IDO mRNA levels were observed at 3 weeks (1.65-fold decrease, *P* = 0.003, Student's *t* test) in NBD STRI compared to levels in neonatal mock-infected STRI (Fig. 3C). The absolute levels of IDO transcripts were particularly low in the HC and CBLM of NBD rats and neonatal mock-infected rats (under 200 copies in HC and under 50 copies in CBLM). STRI had higher levels of IDO mRNA than the HC and CBLM; however, increased levels of IDO were not observed at any time point in NBD STRI.

Inflammation can influence IDO expression (52). As NBD develops in the absence of gross inflammatory cell infiltration in the CNS, we sought to contrast IDO expression in the NBD model with that in the adult infection model, which is characterized by a severe meningoencephalitis. Therefore, we also evaluated IDO mRNA levels by real-time PCR in adult BDV-infected CBLM and hemispheres at 1, 2, 3, and 4 weeks p.i. IDO levels were increased at 2 weeks (9.96-fold increase; *P* = 0.027, Student's *t* test) and 3 weeks (14.97-fold increase; *P* = 0.021, Student's *t* test) in adult BDV-infected CBLM compared to levels in adult mock-infected CBLM and remained elevated in 4-week BDV-infected CBLM (Fig. 3D). In adult BDV-infected hemispheres, IDO levels were decreased at 1 week (2.5-fold decrease *P* = 0.0152, Student's *t* test) and were significantly increased at 2 weeks (5.25-fold increase; *P* = 0.021, Student's *t* test) and 3 weeks (4.36-fold increase; *P* = 0.003, Student's *t* test) compared to levels in adult mock-infected hemispheres, and levels remained elevated in 4-week BDV-infected hemispheres (Fig. 3E).

### Quantitation of KATII transcripts in NBD and adult BDV-infected rat brains.

**FIG 4:**
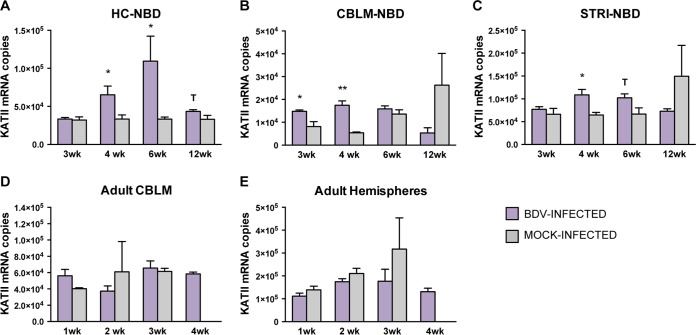
KATII mRNA expression in NBD and adult BDV-infected rat brains by real-time PCR. (A to C) KATII expression at 3, 4, 6, and 12 weeks in NBD and neonatal mock-infected rat HC, CBLM, and STRI. (D and E) KATII expression at 1, 2, 3, and 4 weeks in adult BDV-infected and adult mock-infected rat CBLM and hemispheres. Note that at 4 weeks in the adult model only BDV-infected rats were assessed. *, *P* < 0.05; **, *P* < 0.01; ***, *P* < 0.001; T, *P* < 0.10 (Student's *t* test).

Kynurenine amino transferase II (KATII) is considered to be the major biosynthetic enzyme of kynurenic acid (KYNA) in the mammalian brain (31). In order to evaluate the effects of BDV infection *in vivo* on KATII expression, we quantitated KATII transcripts in HC, CBLM, and STRI of NBD rats at the age of 3, 4, 6, and 12 weeks. KATII transcript levels were increased in NBD HC at 4 weeks (1.96-fold increase; *P* = 0.050, Student's *t* test) and 6 weeks (3.29-fold increase; *P* = 0.020, Student's *t* test) compared to levels in neonatal mock-infected HC (Fig. 4A). KATII transcript levels were also increased at 3 weeks (1.81-fold increase; *P* = 0.018, Student's *t* test) and 4 weeks (3.22-fold-increase; *P* = 0.001, Student's *t* test) in NBD CBLM compared to levels in neonatal mock-infected CBLM (Fig. 4B). Increased KATII mRNA levels were observed at 4 weeks (1.68-fold increase; *P* = 0.017, Student's *t* test) in NBD STRI compared to levels in neonatal mock-infected STRI (Fig. 4C).

We also evaluated KATII mRNA levels by real-time PCR in adult BDV-infected CBLM and hemispheres at 1, 2, 3, and 4 weeks p.i. KATII levels were not altered in adult BDV-infected CBLM compared to levels in adult mock-infected CBLM or in adult BDV-infected hemispheres compared to levels in adult mock-infected hemispheres at 1, 2, and 3 weeks p.i., and 4-week levels in adult BDV-infected rats were similar to those of earlier time points (Fig. 4D and E). These results suggest inherent differences in the regulation of KATII between NBD and adult infection models.

### Quantitation of KMO transcripts in NBD and adult BDV-infected rat brains.

**FIG 5:**
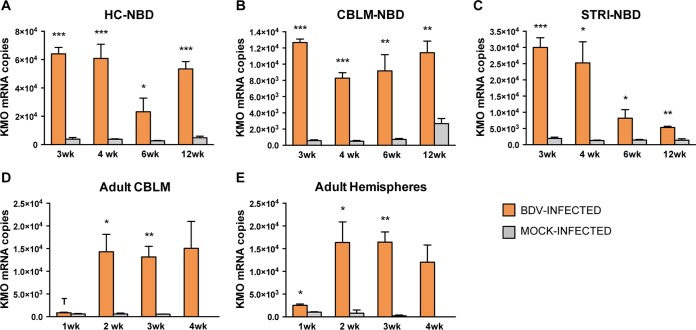
KMO mRNA expression in NBD and adult BDV-infected rat brains by real-time PCR. (A to C) KMO expression at 3, 4, 6, and 12 weeks in NBD and neonatal mock-infected rat HC, CBLM, and STRI. (D and E) KMO expression at 1, 2, 3, and 4 weeks in adult BDV-infected and adult mock-infected rat CBLM and hemispheres. Note that at 4 weeks in the adult model only BDV-infected rats were assessed. *, *P* < 0.05; **, *P* < 0.01; ***, *P* < 0.001; T, *P* < 0.10 (Student's *t* test).

In order to determine whether *in vivo* infection leads to persistent kynurenine-3-monooxygenase (KMO) mRNA induction, we quantitated KMO transcripts in the HC, CBLM, and STRI of NBD rats at the ages of 3, 4, 6, and 12 weeks. KMO transcript levels were increased in NBD HC at 3 weeks (16.67-fold increase; *P* = 0.0001, Student's *t* test), 4 weeks (16.07-fold increase; *P* = 0.0008, Student's *t* test), 6 weeks (8.52-fold increase; *P* = 0.045, Student's *t* test), and 12 weeks (11.10-fold increase; *P* = 0.0005, Student's *t* test) compared to levels in neonatal mock-infected HC (Fig. 5A). KMO transcript levels were also increased at 3 weeks (22.47-fold increase; *P* < 0.0001, Student's *t* test), 4 weeks (16.66-fold increase; *P* < 0.0001, Student's *t* test), 6 weeks (12.88-fold increase; *P* = 0.002, Student's *t* test), and 12 weeks (4.28-fold increase; *P* = 0.004, Student's *t* test) in NBD CBLM compared to levels in neonatal mock-infected CBLM (Fig. 5B). Increased KMO mRNA levels were observed at 3 weeks (15.1-fold increase; *P* = 0.0005, Student's *t* test), 4 weeks (19.3-fold increase; *P* = 0.049, Student's *t* test), 6 weeks (5.62-fold-increase; *P* = 0.029, Student's *t* test), and 12 weeks (3.87-fold increase; *P* = 0.001, Student's *t* test) in NBD STRI compared to levels in neonatal mock-infected STRI (Fig. 5C).

In order to determine whether BDV infection has any differential impact on KMO expression in NBD rats compared to that in adult BDV-infected rats, we also evaluated KMO mRNA levels by real-time PCR in adult BDV-infected CBLM and hemispheres at 1, 2, 3, and 4 weeks p.i. KMO levels were increased at 2 weeks (23.35-fold increase; *P* = 0.037, Student's *t* test) and 3 weeks (23.29-fold increase; *P* = 0.008, Student's *t* test) in adult BDV-infected CBLM compared to levels in adult mock-infected CBLM and remained elevated at 4 weeks in BDV-infected adult rats. KMO levels also tended to be higher at 1 week p.i. in BDV-infected CBLM but did not reach significance (Fig. 5D). In adult BDV-infected hemispheres, KMO levels were increased at 1 week (2.47-fold increase; *P* = 0.014, Student's *t* test), 2 weeks (20.40-fold increase; *P* = 0.043, Student's *t* test), and 3 weeks (86.1-fold increase; *P* = 0.002, Student's *t* test) compared to levels in adult mock-infected hemispheres and remained elevated at 4 weeks in adult BDV-infected hemispheres (Fig. 5E).

### Immunolocalization of KMO in NBD rat brains.

**FIG 6:**
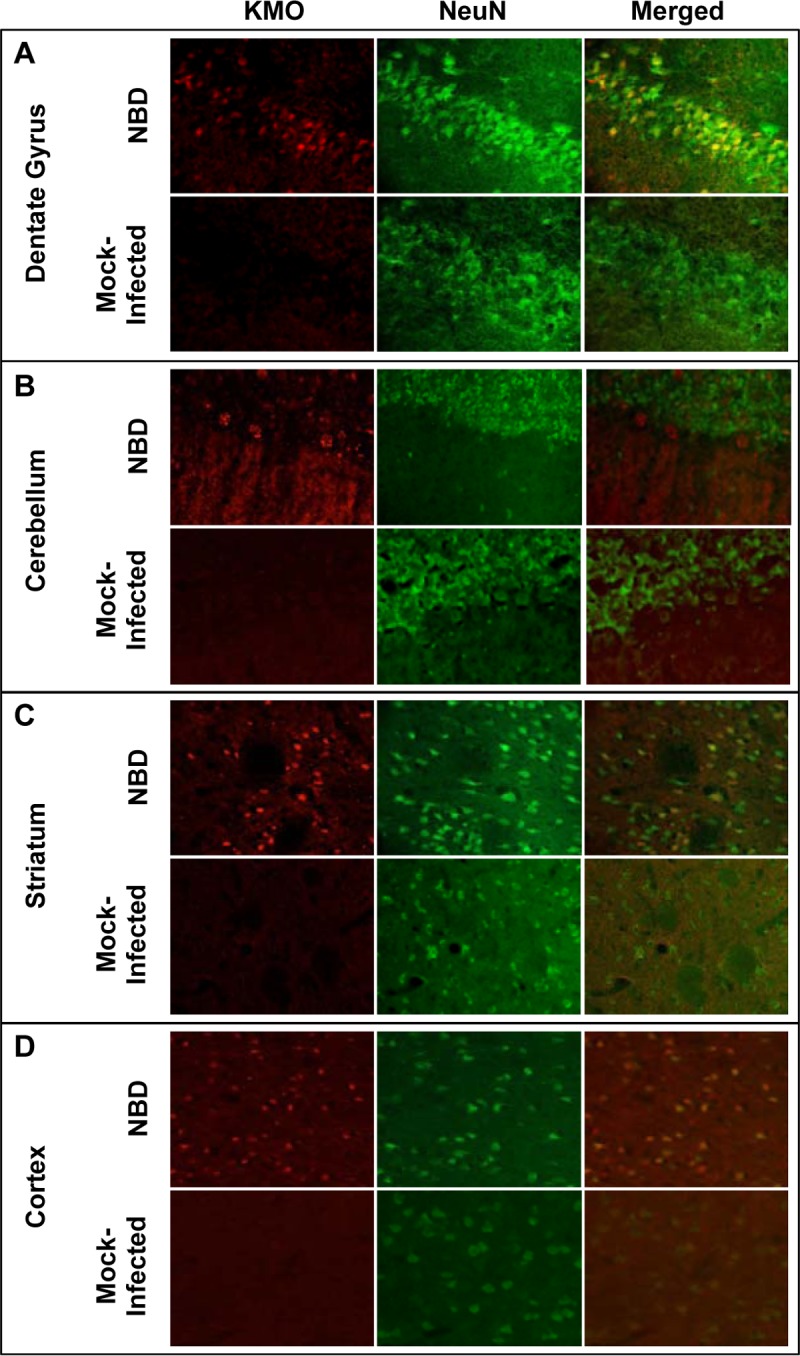
Immunofluorescence localization of KMO in NBD rat brains. Anti-KMO fluorescence in dentate gyrus of the HC (A), CBLM (B), STRI (C), and cortex (D) is shown in red in NBD and neonatal mock-infected rats. The neuronal marker anti-NeuN (green) was used for colocalization of KMO with neurons. Merged images show colocalization of KMO and NeuN fluorescence (yellow).

Our findings of dramatically increased KMO mRNA in NBD HC, CBLM, and STRI led us to investigate the distribution and localization of KMO protein in NBD rat brains compared to that in neonatal mock-infected rat brains. Double-label immunofluorescence, using antibodies (Abs) to KMO and a neuron-specific marker (NeuN), was performed in dentate gyrus of HC, CBLM, STRI, and cortex (Fig. 6A to D). Anti-KMO fluorescence was prominent in NBD rat dentate gyrus, CBLM, STRI, and cortex compared to results in corresponding brain regions in neonatal mock-infected rats. Thus, increased levels of KMO mRNA were attended by increased levels of KMO protein in NBD rat brains. Strong KMO fluorescence colocalized with the neuronal marker NeuN in dentate gyrus granule cell neurons, as well as in neurons in the STRI and cortex of NBD rat brains (Fig. 6A, C, and D). Punctate staining for KMO was evident in cerebellar Purkinje neurons in NBD rats. Because cerebellar Purkinje neurons are not labeled by NeuN, NeuN did not colocalize with KMO; however, these neurons are readily distinguished based on their size and distribution at the boundary of the granule cell layer of the cerebellum (Fig. 6B). Collectively, these data suggest that KMO protein expression is induced in neurons throughout the brains of NBD rats.

### Quinolinic acid and l-kynurenine levels in NBD rat brains.

**FIG 7:**
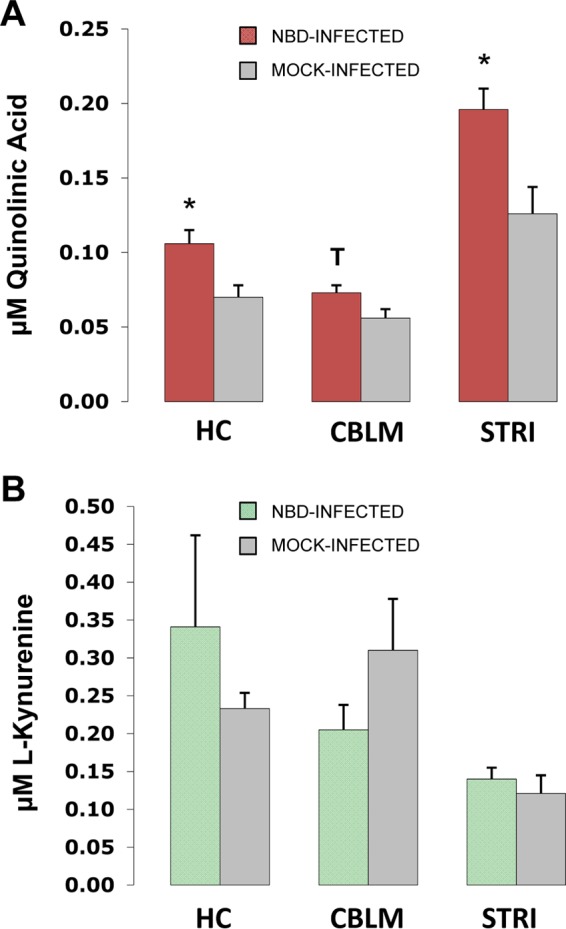
Quinolinic acid and l-kynurenine levels in NBD rat brains. Concentrations are shown for QUIN (A) and l-KYN (B) in HC, CBLM, and STRI of NBD (*n* = 7) and mock-infected (*n* = 5) rats at 4 weeks. *, *P* < 0.05; T, *P* < 0.10 (Student's *t* test).

As levels of KMO were highly increased in NBD and as KMO is responsible for conversion of l-kynurenine (l-KYN) along the pathway leading to the production of the excitotoxin QUIN, we further evaluated QUIN and l-KYN levels in NBD and neonatal mock-infected rat brains at 4 weeks p.i. Levels of QUIN were significantly increased in HC (1.51-fold increase; *P* = 0.019, Student's *t* test) and STRI (1.55-fold increase; *P* = 0.011, Student's *t* test) of NBD rats compared to levels in neonatal mock-infected rats. While not significant, a trend toward increased QUIN levels was found in the NBD CBLM compared to levels in neonatal mock-infected CBLM (*P* = 0.070, Student's *t* test) (Fig. 7A). We also evaluated l-KYN levels; differential levels of l-KYN were not observed in NBD HC, CBLM, and STRI compared to levels in neonatal mock-infected HC, CBLM, and STRI (Fig. 7B).

### Quantitation of kynurenine pathway enzyme transcripts in persistently infected C6 astroglioma cells.

**FIG 8:**
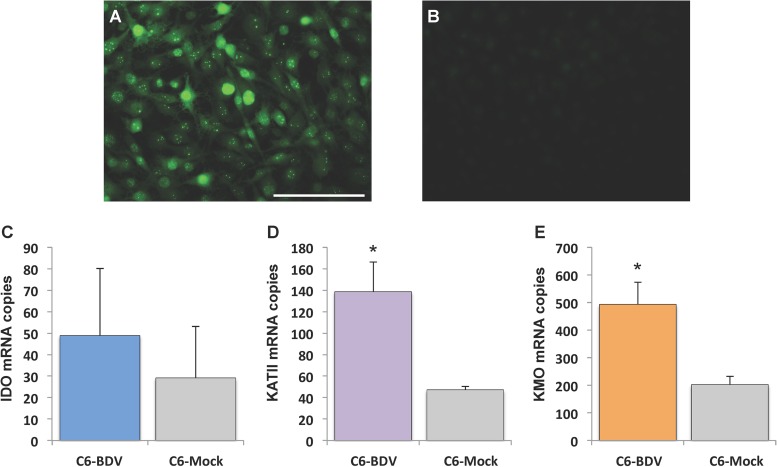
Quantitation of IDO, KATII, and KMO mRNA expression in C6-BDV cells. Immunofluorescence staining with anti-BDV p40 (nucleoprotein) in persistently infected C6-BDV cells (A) versus that in C6-mock cells (B) is shown. Note the prominent staining in C6-BDV cells, including characteristic punctate staining in the nucleus. (C to E) Real-time PCR comparing mRNA expression of IDO, KATII, and KMO in C6-BDV cells and C6-mock cells. *, *P* < 0.05 (Student's *t* test).

Given our findings with *in vivo* models demonstrating altered expression of kynurenine pathway enzymes, we pursued real-time PCR assays targeting IDO, KATII, and KMO in cultures of persistently BDV-infected C6 (C6-BDV) cells and C6 noninfected control (C6-mock) cells in order to evaluate direct *in vitro* effects of BDV infection in cell culture. Persistent infection of C6-BDV cells at passage 15 was confirmed by immunofluorescence for BDV antigen (Fig. 8A); immunofluorescence in C6-mock cells is shown for comparison (Fig. 8B). IDO gene expression levels did not differ between C6-BDV and C6-mock cells (Fig. 8C). In contrast, transcript levels in C6-BDV cells were higher for KATII (Fig. 8D) (2.94-fold increase; *P* = 0.030, Student's *t* test) and KMO (Fig. 8E) (2.43-fold increase; *P* = 0.027, Student's *t* test).

## DISCUSSION

In this study, we investigated enzymes of the kynurenine pathway of tryptophan degradation in brains from NBD and adult BDV-infected rats, as well as in BDV-infected cultured C6 cells. Tryptophan degradation along the kynurenine pathway mediates the production of the neuroactive metabolites KYNA, 3-HK, and QUIN, which are implicated in several neurodegenerative pathologies (39). Thus, differential regulation of the kynurenine pathway could contribute to the neuropathologic changes observed in BDV-infected rat brains.

Here, we quantitated levels of IDO, KATII, and KMO mRNAs *in vivo* in both NBD and adult BDV-infected rat brains. In rats infected with BDV as adults, we focused on the time interval corresponding to the acute phase of inflammation, during which time a severe meningoencephalitis develops, and the immune system intervention is prominent (53). We evaluated gene expression changes up to 12 weeks in NBD rats in order to determine whether long-term upregulation of kynurenine pathway enzymes was evident and extended well into adulthood.

Dysregulation of IDO, KATII, and KMO mRNA levels was observed in both models of infection compared to levels in the controls. However, distinct differences in gene expression levels for these kynurenine pathway enzymes were observed between the adult and the NBD models of infection. In adult CBLM, BDV infection dramatically increased IDO transcript levels at 2 and 3 weeks p.i., and transcripts remained elevated at 4 weeks p.i. Adult hemispheres showed an initial inhibition of IDO expression (1 week p.i.), followed by significant overexpression at later time points (2 and 3 weeks p.i.). IDO is the rate-limiting enzyme in the kynurenine pathway, thus determining the metabolic fate of tryptophan. IDO may be actively expressed in infiltrating immune cells or indirectly induced in resident cells of the CNS through the action of cytokines (54). The prominent and sustained upregulation of IDO transcripts detected in adult BDV-infected rat brains may thus derive from the immune response to viral infection in the CNS. In fact, the acute phase of infection in the adult model occurs approximately 2 to 4 weeks p.i. and is characterized by infiltrating CD4^+^, CD8^+^, and plasma cells (53). Consistent with the timing of these events, we found high levels of IDO mRNA in both the CBLM and hemispheres beginning at 2 weeks p.i. and continuing to at least 4 weeks. The increased levels of IDO in the adult rats did not correlate with the progressive increase in viral burden from 1 to 4 weeks but, rather, reached high levels by 2 weeks and remained highly elevated thereafter. Furthermore, quantitation of gene expression showed higher expression levels of KMO and KATII in C6-BDV cells while IDO levels remained undisturbed. This result is consistent with literature on IDO induction suggesting that transcriptional upregulation occurs in response to inflammatory stimuli, with gamma interferon acting as the primary inducer (39, 55). These results suggest that the neuroinflammatory response to BDV infection in rats infected as adult likely contributes more substantively to increased IDO levels, which may further drive the kynurenine pathway toward enhanced production of neurotoxic metabolites (56). Accordingly, we speculate that the massive destruction of neurons in the adult infection model may result from synergy between infiltrating immune cells and direct BDV-induced changes in kynurenine pathway enzymes in CNS-resident cells.

The equilibrium between KYNA, produced by the enzyme KATII, and QUIN, produced by the enzyme KMO, plays an important role in the pathophysiology of the nervous system (39). In the adult CBLM and hemispheres, KATII mRNA levels were not significantly altered by BDV infection. This finding is somewhat surprising, given that BDV appears to induce KATII *in vitro*. However, this finding is consistent with previous reports demonstrating that brain KATII expression is not impacted by inflammatory stimuli (57, 58).

KMO enzymatic activity leads toward production of 3-HK and QUIN. Dysfunction of KMO expression is associated with a broad range of neuropathologic findings, including those of Alzheimer's disease and Huntington's disease (59, 60). KMO levels were dramatically increased in BDV-infected adult rat CBLM and hemispheres as early as 1 to 2 weeks p.i., with extreme elevation at 2, 3, and 4 weeks (up to an 86.1-fold increase). This pattern was similar to IDO expression and did not correlate with the progressive increase in viral burden. Similar to expression of IDO, induction of brain KMO expression is reported to arise as a result of inflammatory challenge (52). As BDV alone does not seem to influence IDO expression *in vitro*, inflammatory mediators in the adult model may be solely responsible for IDO induction. In contrast, BDV does upregulate the expression of KMO in NBD and *in vitro*. As such, compounding effects of direct BDV-induced and inflammation-induced regulation may contribute to the dramatic dysregulation of KMO in the adult infection model.

Patterns were distinctly different in the NBD model. In NBD, IDO mRNA was moderately increased at specific postnatal time points in the HC and CBLM, but not in the STRI. Only after 1 month of age do rodent immune systems reach full maturation with a potential to efficiently respond to antigens (61). Early-life BDV infection in NBD occurs within this window of immaturity, resulting in a tolerant infection generally devoid of immune cell infiltration. These differences in immunopathogenesis likely explain the different patterns of transcriptional expression of IDO between the adult and NBD models. Nonetheless, IDO induction is detected at specific time points in NBD although expression levels are much lower than in the adult model. A fleeting inflammatory reaction in the CNS can occur at 4 to 5 weeks in NBD (12). In the NBD HC, IDO levels were significantly increased only at the 4- and 6-week time points; in NBD CBLM, IDO levels were increased only at the 4-week time point. Thus, transient inflammation may be responsible for modest, temporally restricted increases in IDO transcription in NBD brain regions.

In contrast to the adult model where no differences were found in KATII expression levels, in NBD KATII mRNA levels were increased at 4 weeks p.i. in the HC, CBLM, and STRI. In addition, KATII was increased at 6 weeks in the HC and at 3 weeks in the CBLM but was not significantly altered at any other time points in any of the three brain regions examined.

Most remarkably, we show a persistent induction of KMO mRNA levels in NBD HC, CBLM, and STRI at all time points (3, 4, 6, and 12 weeks). Although l-KYN metabolism within the brain is thought to be predominantly confined to glia (39), KMO protein levels, based on immunofluorescence, were clearly higher in NBD neuronal cells, which are the primary targets of BDV infection, and KMO transcript levels did not correlate with the levels of astrogliosis as assessed by GFAP quantitation. Additionally, elevated levels of KMO have been observed *in vitro* in primary neuron cultures infected with neurotropic influenza virus and in brain in neonatal rodent infection with neurotropic influenza virus (37). These findings suggest that KMO induction in NBD rat brains is a consequence of direct neuronal infection rather than of the proliferation and infiltration of macrophages, microglia, and astrocytes that are hallmarks of the model (12).

To evaluate whether disturbances in kynurenine pathway enzymes in NBD rats resulted in alterations of kynurenine pathway metabolites, we measured brain levels of l-KYN and QUIN. Although IDO mRNA levels were increased at 4 weeks in HC and CBLM, our results show that l-KYN levels were not significantly increased in NBD rats at 4 weeks in HC, CBLM, or STRI. However, elevated KMO expression in NBD HC, CBLM, and STRI at 4 weeks would be expected to shift metabolism of l-KYN toward QUIN production. Thus, elevated production of l-KYN resulting from IDO induction may be balanced by increased expression of KMO, which would increase the rate of conversion of l-KYN to QUIN. Our results show that QUIN levels were increased in NBD at 4 weeks in HC and STRI. A trend was observed only in CBLM. That the absolute IDO and KMO mRNA levels detected in CBLM were significantly lower than levels in HC and STRI may help explain why CBLM QUIN differences were less prominent. However, the relationship between mRNA levels, protein levels, and overall flux through the pathway cannot be readily assessed in these *in vivo* models and will require more extensive *in vitro* studies. Furthermore, neurons in the HC and STRI are particularly sensitive to the detrimental effects of QUIN (62, 63). While we have not examined levels of 3-HK, this metabolite could further contribute to neural damage through its ability to generate hydroxyl radicals if it is also elevated by BDV infection. Altered expression of kynurenine enzymes and metabolites could also be linked to previous findings demonstrating that the metabolism of several amino acids, including tryptophan, were altered following BDV infection in oligodendroglia (64).

Although the mechanisms leading to neuropathologic disturbances in neonatal BDV are still poorly understood, we speculate that the overexpression of KMO and the associated increase in production of QUIN might underlie neuronal injury and loss. QUIN is a potent endogenous neurotoxin and can promote apoptosis in neurons, oligodendrocytes, and astrocytes (33, 65). QUIN toxicity in neurons is triggered by overstimulation of NMDA receptors, resulting in disruption of intracellular Ca^2+^ homeostasis and the formation of free radicals (66–68). Neuronal excitotoxicity induced *in vivo* by QUIN is associated with impairment of sarcoplasmic/endoplasmic reticulum Ca^2+^-ATPase (SERCA) activity that may contribute to disturbances in Ca^2+^ homeostasis (69). The endoplasmic reticulum is sensitive to disruption of calcium homeostasis (70). We have previously shown that BDV infection of newborn rats leads to endoplasmic reticulum stress in neurons and astrocytes and reduced expression of SERCA mRNA in the brain (17). Thus, QUIN-induced excitotoxicity and its downstream impact on SERCA and Ca^2+^ homeostasis may serve as the impetus for disturbances in endoplasmic reticulum function in NBD rats. Also, QUIN excitotoxicity generates free radicals that mediate oxidative DNA damage and stimulate poly(ADP-ribose) polymerase 1 (PARP-1) activity. Activation of PARP-1 depletes neuronal NAD^+^ and ATP reservoirs, leading to apoptosis (71, 72). We previously demonstrated PARP-1 activation in NBD rat brains (21). Our current findings demonstrating elevated mRNA expression of kynurenine pathway enzymes and QUIN levels suggest that QUIN could also serve as the upstream stimulus for activation of PARP-1, with consequent neuronal cell loss in NBD rat brains. Furthermore, QUIN can increase glutamate release and inhibit its reuptake by astrocytes (73, 74). Intriguingly, glutamate uptake in feline cortical astrocytes is inhibited by BDV infection, and extracellular levels of striatal glutamate are increased in the NBD rat model (75–77). As such, QUIN-induced alteration of glutamate concentration in the synaptic space could further contribute to QUIN toxicity and neuropathology. However, asymptomatic BDV infection in horses and infection of cortical neurons and rats with strains of BDV different from the strain used in our study have been associated with lower levels of glutamate (78, 79). Thus, it remains unclear as to whether impacts on glutamate are discordant in BDV infection depending on the host species and virus strain evaluated.

While BDV establishes a persistent, noncytolytic infection in neurons and other cells *in vitro*, BDV infection results in massive neuronal loss *in vivo*, most prominently in HC and CBLM in the NBD model. The mechanisms contributing to these discordant pathological outcomes of BDV infection are unclear. Activation of the kynurenine pathway could provide a plausible explanation for these discordant outcomes. KMO inhibition has been shown to ameliorate the outcome of several neurological disorders (31, 80), and increased levels of QUIN are reported in suicidal patients and patients with severe depression (81, 82). In contrast, KMO activity is reduced in the prefrontal cortex in schizophrenia, with associated shifts in the kynurenine pathway toward the production of KYNA (83). Thus, despite the neuroprotective role of KYNA, differential dysregulation of the kynurenine pathway favoring overproduction of either QUIN or KYNA may contribute to different pathologies. Future studies using specific and selective KMO inhibitors, such as UPF648, could further clarify the role of QUIN in the onset of neuropathologic changes in models of BDV infection (84, 85).

The present results show the relationship between BDV infection and altered brain expression of enzymes of the kynurenine pathway of tryptophan degradation in NBD and adult BDV-infected rats. In the former, increased KMO expression correlates with increased production of QUIN, a potential mediator of the neurotoxic effects of BDV. Excitotoxicity, reactive oxygen species-mediated oxidative stress, kynurenine pathway dysregulation, and increased QUIN levels are commonly reported in human neurodevelopmental and neurodegenerative disorders. While several studies have reported serological or molecular findings implicating BDV in human neuropsychiatric disorders, including schizophrenia and mood disorders (86–90), these findings remain controversial as other studies failed to find such associations and suggest that contamination with laboratory virus strains or assay specificity may have confounded some analyses (91–93). More recent work has found the related, but distinct, variegated squirrel 1 bornavirus (VSBV-1) associated with fatal human cases of encephalitis (94). Regardless of the validity of a link between human BDV exposures and human neuropsychiatric disease, the NBD and adult BDV experimental models may provide a deeper understanding of the mechanisms leading to neurodevelopmental and neurodegenerative abnormalities, with particular relevance to human conditions associated with kynurenine pathway dysregulation.

## MATERIALS AND METHODS

### Ethics statement.

All rats were handled in accordance with the guidelines of the Association for Assessment and Accreditation of Laboratory Animal Care International, with the approval of the Institutional Animal Care and Use Committee at Columbia University under approval ID AC-AAAA0182.

### Cells, animals, and virus inoculation.

C6 astroglioma cells (ATCC CCL107) were grown in Dulbecco's modified Eagle medium containing penicillin, streptomycin, 1% glutamine, and 10% heat-inactivated fetal bovine serum. C6 cells were infected with the Giessen strain He/80 of BDV from infected rat brain homogenate in 25-cm^2^ tissue culture flasks. At 3 days p.i., cells were trypsinized and passaged (1:4) to establish persistently BDV-infected C6 (C6-BDV) cells. Thereafter, C6-BDV cells were passaged (1:4) every 3 to 4 days under the same conditions as noninfected control (C6-mock) cells for 15 additional passages. Lewis rat dams were obtained from Charles River Laboratories (Wilmington, MA, USA). Within 12 h of birth, Lewis rat pups were inoculated into the right cerebral hemisphere with 50 μl of 5 × 10^3^ tissue culture infectious doses (TCID_50_) of BDV strain He/80 (NBD) or phosphate-buffered saline (PBS) (control; mock infection). NBD and mock-infected rats were sacrificed at postnatal days (PND) 21 (3 weeks; *n* = 4 NBD rats, *n* = 4 mock-infected rats), 28 (4 weeks; *n* = 7 NBD rats, *n* = 5 mock-infected rats), 42 (6 weeks; *n* = 5 NBD, *n* = 5 mock-infected rats), and 84 (12 weeks; *n* = 4 NBD, *n* = 3 mock-infected rats) for dissection and RNA analysis. Adult Lewis rats were infected at 5 weeks of age by injection of 5 × 10^3^ TCID_50_ of BDV strain He/80 into the left hemisphere or inoculated with phosphate-buffered saline (mock infection). Adult BDV- and mock-infected rats were sacrificed at p.i. days 7 (1 week; *n* = 6 adult BDV-infected rats, *n* = 3 mock-infected rats), 14 (2 weeks; *n* = 5 adult BDV-infected rats, *n* = 3 mock-infected rats), 21 (3 weeks; *n* = 6 adult BDV-infected rats, *n* = 3 mock-infected rats), and 28 (4 weeks; *n* = 4 adult BDV-infected rats) for dissection and RNA analysis. Mock-infected rats at p.i. day 28 were not included. Expression levels of genes in adult BDV-infected rats at 4 weeks p.i. were included to compare expression levels of genes during BDV infection at this time point with levels of genes observed at earlier time points (1, 2, and 3 weeks). No adult BDV-infected rats died as a result of disease during the 28-day course of this study, consistent with previous reports demonstrating a high rate of mortality occurring only at later time points p.i. in the adult model (between 1 and 4 months p.i.) (45).

### RNA extraction.

RNA from C6-mock or C6-BDV astroglioma cells was extracted after cells were washed with PBS from two 25-cm^2^ flasks, each with TRIzol, according to the manufacturer's protocol (Invitrogen, Carlsbad, CA, USA). NBD rats and age-matched, mock-infected rats were terminally anesthetized with CO_2_ at PND 21, 28, 42, and 84, and hippocampus (HC), cerebellum (CBLM), and striatum (STRI) were immediately dissected, snap-frozen in TRIzol, and extracted according to the manufacturer's protocols. Adult BDV- and mock-infected rats were terminally anesthetized with CO_2_ at p.i. days 7, 14, 21, and 28, and hemispheres and CBLM were immediately dissected, snap-frozen in TRIzol, and extracted. RNA concentrations and integrity were determined using a NanoDrop ND-1000 spectrophotometer (NanoDrop Technologies, Wilmington, DE, USA) and Bioanalyzer (Agilent Technologies, Foster City, CA, USA) and stored at −80°C.

### Quantitative real-time PCR.

Gene-specific PCR primers and fluorophore-labeled probes specific for rat IDO, KATII, KMO, and porphobilinogen deaminase (PBGD) as a housekeeping gene control were designed for real-time PCR, using Primer Express, version 1.0, software (Applied Biosystems, Foster City, CA, USA) (Table 1). Probes were labeled with a 5′-end fluorescent reporter dye (6-carboxyfluorescein) and a 3′-end quencher dye (6-carboxytetramethylrhodamine). BDV phosphoprotein (BDV P) RNA and rat glial fibrillary acidic protein (GFAP) mRNA transcripts were quantitated using previously described assays (95, 96) (Table 1). Determination of target transcript copy number was performed as previously described (18). RNA from HC, CBLM, and STRI of individual NBD or neonatal mock-infected rats or RNA from CBLM and hemispheres of individual adult BDV-infected or adult mock-infected rats was used for real-time PCR assays. cDNA was synthesized, using TaqMan reverse transcription reagents (Applied Biosystems), from 2 μg of RNA per 100-μl reaction mixture from the HC, CBLM, and STRI of either individual NBD rats (3 weeks, *n* = 3 to 4 NBD rats; 4 weeks, *n* = 7 NBD rats; 6 weeks, *n* = 4 to 5 NBD) or neonatal mock-infected rats (3 weeks, *n* = 3 to 4 mock-infected rats; 4 weeks, *n* = 4 to 5 mock-infected rats; 6 weeks, *n* = 3 to 5 mock-infected rats) or from the CBLM and hemispheres of either individual adult BDV-infected rats (1 week, *n* = 5 to 6 adult BDV-infected rats; 2 weeks, *n* = 4 to 5 adult BDV-infected rats; 3 weeks, *n* = 3 to 6 adult BDV-infected rats; 4 weeks, *n* = 3 to 4 adult BDV-infected rats) or adult mock-infected rats (1 week, *n* = 3 mock-infected rats; 2 weeks, *n* = 3 mock-infected rats; 3 weeks, *n* = 3 mock-infected rats). Each sample was assayed in duplicate. Each 25-μl amplification reaction mixture contained 10 μl of template cDNA, 12.5 μl of universal master mix (Applied Biosystems) or SYBR green PCR master mix (Applied Biosystems), 200 nM probe (except for the GFAP assay, which was a SYBR green assay), and 300 nM gene-specific primers. Thermocycling conditions using a model 7700 sequence detector system (Applied Biosystems) consisted of the following: stage 1, 1 cycle at 50°C for 2 min; stage 2, 1 cycle at 95°C for 10 min; and stage 3, 45 cycles at 95°C for 15 s and 60°C for 1 min. A PBGD fragment was amplified in duplicate reactions by real-time PCR on the same plate as the gene of interest, and the mean concentration of PBGD in each sample was used to normalize values of target gene expression. The final results were expressed as the normalized mean number of copies per 200 ng of total RNA for IDO, KMO, KATII, BDV P, and GFAP.

**TABLE 1:** Primer and probe sequences for quantitative PCR assays

Gene (GenBank accession no.)	Primer or probe^*a*^	Sequence (5′–3′)^*b*^	Reaction concn (nM)	Amplicon size (bp)
IDO (NM_023973.1)	For	GGATGCGTGACTTCGTGGAT	300	100
	Rev	GTACAGCAGACCCTCCGGC	300	
	Probe	FAM-TCTTCGCATATATTTGTCTGGTTGGAAGGGC-TAMRA	200	
KMO (NM_021593)	For	TCTCGGGAAAGAAGTCTGCAA	300	100
	Rev	CTCCACGGCAGTCAGCAGAT	300	
	Probe	FAM-TGGGAACAAGTCACAGTATATCCTTTCAATAAGCAGA-TAMRA	200	
KATII (NM_017193.1)	For	AGTGATCTGGGAAGCCGTTCT	300	100
	Rev	AGGCTCGTTGCAGTGAGGAA	300	
	Probe	FAM-TCCACGCGACCAGCAGAGACATGA-TAMRA	200	
PBGD (X06827)	For	ATTCGGGGAAACCTCAACACC	300	157
	Rev	CTGACCCACAGCATACATGCAT	300	
	Probe	FAM-GCAAGATCTGGCCCACCCGGTT-TAMRA	200	
BDV P (AJ311522)	For	GAACCCCTCCATGATCTCAGAY	300	88
	Rev	CTCYGTCACTAGCTTCTTGATRAG	300	
	Probe	FAM-CAGCGAACCGGAAGGGAGCAGCTATC-BHQ1	200	
GFAP (NM_017009.2)	For	CAGACTTTCTCCAACCTCCAG	300	138
	Rev	CTCCTGCTTCGAGTCCTTAATG	300	

a For, forward; Rev, reverse.

b FAM, 6-carboxyfluorescein; TAMRA, 6-carboxytetramethylrhodamine; BHQ1, Black Hole quencher 1.

### Histological analysis and immunofluorescence.

C6-BDV and C6-mock cells were grown in glass chamber slides and fixed with 4% paraformaldehyde in phosphate-buffered saline (PBS), followed by incubation for 10-min at room temperature in PBS containing 1% Triton X-100. After three washes with PBS, cells were blocked with 1% normal goat serum for 1 h at room temperature, followed by staining with rabbit anti-BDV P40 (1:5,000) in blocking solution for 1 h. After cells were washed in PBS, they were stained with secondary antibody, Cy2-conjugated anti-rabbit IgG (diluted 1:200 in PBS; Jackson ImmunoResearch Laboratories, Inc., West Grove, PA, USA), for 45 min at 37°C. Finally, slides were washed extensively with PBS and mounted in 50% glycerol. Under CO_2_ anesthesia, 4-week-old NBD (*n* = 5/age group) and mock-infected (*n* = 4/age group) rats were perfused via left ventricular puncture with PBS (1 ml/g body weight), followed by buffered 4% paraformaldehyde (1 ml/g body weight). Brains were postfixed in 4% paraformaldehyde overnight at 4°C and cryoprotected with graded sucrose solutions. Cryostat sections (14 μm) were collected onto glass slides (Super Frost Plus; Fisher Scientific, Pittsburgh, PA, USA). Double-label immunofluorescence microscopy was carried out as previously described (20), using the following primary antibodies: rabbit anti-KMO Ab (1:50; Proteintech, Chicago, IL, USA) and mouse anti-neuronal nuclei (NeuN) monoclonal antibody (MAb) (1:100; Chemicon International, Temecula, CA, USA). Secondary antibodies were Cy3-conjugated anti-rabbit IgG (1:200; Jackson ImmunoResearch) and Cy2-conjugated anti-mouse IgG (1:200; Jackson ImmunoResearch).

### Analysis of QUIN and l-KYN content in brain.

NBD rats (*n* = 7) and mock-infected rats (*n* = 5) were anesthetized at PND 28 with CO_2_ and rapidly decapitated, and brains were removed for regional dissections over ice. Tissues representing HC, CBLM, and STRI were dissected from 2-mm coronal sections, placed in separate, tared Eppendorf tubes, immediately frozen on dry ice, and then stored at −70°C until further processing. Frozen tissue samples were homogenized in 250 μl of 0.1 N perchloric acid and centrifuged. Fifty-microliter aliquots of the supernatant were supplemented with 10 μl of internal standard solution (1-methyl-tryptophan [1-Me-TRYP] and l-glutamic acid 5-methyl ester [Glu-O-Me]; 100 nM final concentration), injected onto a polyhydroxyethyl A hydrophilic interaction liquid chromatography (HILIC) column (200 by 2 mm; 3-μm particle size; 10-nm pore size; PolyLC, Inc., Columbia, MD, USA), and eluted using an 85 to 20% acetonitrile gradient over 20 min (the mobile phase contained 20 mM ammonium formate and 0.1% formic acid; 150-μl/min flow rate). Analytes and internal standards were detected by electrospray ionization mass spectrometry using selected ion monitoring in positive ion mode (QUIN *m/z* = 168.2; l-KYN *m/z* = 209.2; 1-Me-TRYP *m/z* = 219.2; Glu-O-Me *m/z* = 162.2) [1100 SL MSD column; Agilent Technologies]). Analyte concentrations were normalized to the sample protein content as determined from the tissue pellets.

### Statistical analysis.

The significance of observed differences between NBD and control groups was assessed by Student's *t* tests for real-time PCR and metabolite analysis. Analysis was carried out using StatView software (version 5.0.1; SAS Institute, Inc., Cary, NC, USA). Values were considered to be significant at a *P* value of <0.05.
